# Eye movement as a biomarker of impaired organizational strategies during visual memory encoding in obsessive–compulsive disorder

**DOI:** 10.1038/s41598-021-97885-1

**Published:** 2021-09-15

**Authors:** Minah Kim, Woncheol Shin, Tak Hyung Lee, Taekwan Kim, Wu Jeong Hwang, Jun Soo Kwon

**Affiliations:** 1 Department of Neuropsychiatry, Seoul National University Hospital, Seoul, Republic of Korea; 2 Department of Psychiatry, Seoul National University College of Medicine, 101 Daehak-no, Chongno-gu, Seoul, 03080 Republic of Korea; 3 Graduate School of Artificial Intelligence, Korea Advanced Institute of Science and Technology, Daejeon, Republic of Korea; 4 Healthcare Sensor Laboratory, Device Research Center, Samsung Advanced Institute of Technology, Samsung Electronics Co., Ltd., Suwon, Republic of Korea; 5 Department of Brain and Cognitive Sciences, Seoul National University College of Natural Sciences, Seoul, Republic of Korea

**Keywords:** Neuroscience, Psychology, Biomarkers

## Abstract

The symptoms of obsessive–compulsive disorder (OCD) are largely related to impaired executive functioning due to frontostriatal dysfunction. To better treat OCD, the development of biomarkers to bridge the gap between the symptomatic-cognitive phenotype and brain abnormalities is warranted. Therefore, we aimed to identify biomarkers of impaired organizational strategies during visual encoding processes in OCD patients by developing an eye tracking-based Rey–Osterrieth complex figure test (RCFT). In 104 OCD patients and 114 healthy controls (HCs), eye movements were recorded during memorization of the RCFT figure, and organizational scores were evaluated. Kullback–Leibler divergence (KLD) scores were calculated to evaluate the distance between a participant’s eye gaze distribution and a hypothetical uniform distribution within the RCFT figure. Narrower gaze distributions within the RCFT figure, which yielded higher KLD scores, indicated that the participant was more obsessed with detail and had less organizational strategy. The OCD patients showed lower organizational scores than the HCs. Although no group differences in KLD scores were noted, KLD scores were significantly associated with organization T scores in the OCD group. The current study findings suggest that eye tracking biomarkers of visual memory encoding provide a rapidly determined index of executive functioning, such as organizational strategies, in OCD patients.

## Introduction

The characteristic symptoms of obsessive–compulsive disorder (OCD) are obsession and compulsion, which often present as recurrent intrusive thoughts and repetitive behaviors. The inability to control one’s thoughts and behaviors is associated with neurocognitive impairments, which are related with underlying brain dysfunction, such as imbalanced activity in the cortico-striato-thalamo-cortical loop^1–3^. However, similar to many other psychiatric disorders^4,5^, current diagnosis and treatment of OCD are mostly based on the symptomatic-cognitive phenotype (i.e., behavioral level), producing significant heterogeneity within the disorder in both brain dysfunction and treatment response^6–8^. In addition, biomarker studies that intended to bridge the gap between the symptom-cognitive level and the brain level dysfunction of OCD require complex experimental designs and equipment^7–9^. Thus, to achieve a better understanding of the pathophysiology of OCD and to provide better treatment for the patients in clinical practice, investigation of easily determinable biomarkers that directly reflect brain dysfunctions that cause symptom-cognitive phenotypes is warranted.

Among the various domains of neurocognitive dysfunction found in OCD patients, the largest effect sizes have been reported in executive function, including organizational abilities during visual memory encoding as measured by the Rey–Osterrieth Complex Figure Test (RCFT)^10^. OCD patients exhibit dysfunctions in organizational strategies during the initial encoding of the RCFT figure, and this executive dysfunction is critical in impairments of immediate recall^11–13^. Therefore, the RCFT has been widely used to evaluate executive function impairments, which are largely dependent on frontostriatal system function, in OCD patients^11,14^. However, the RCFT measures the cognitive-behavioral level phenotype, which has a less direct relationship with brain dysfunction of OCD than do biomarkers. In addition, the RCFT has several limitations for use in clinical practice: it is an indirect measurement of encoding that is affected by visuomotor function, too time consuming, and too complex in terms of both the task structure and scoring system.

Thus, studies to identify rapidly and easily determinable biomarkers should be developed for use in clinical practice to bridge the gap between the symptomatic-cognitive phenotype and brain abnormalities in OCD. In this regard, eye tracking-based RCFT could provide a potential biomarker related to frontostriatal dysfunction of OCD that offers advantages in clinical utility. Because eye tracking provides information about the location where a person looks, it provides a more direct, simple, and rapid measurement of visual encoding processes, which makes it easy to apply in actual clinical practice. Previous eye tracking studies in OCD patients reported that OCD patients had characteristic uncontrolled and regressive eye movements during reading sentences or words^15–17^. However, those studies had small sample sizes and did not measure organizational abilities during visual memory encoding processes.

In the current study, we aimed to develop an eye tracking-based RCFT and identify a biomarker that is reflective of organizational abilities during visual memory encoding in patients with OCD. First, we expected that OCD patients would show poor immediate recall performances and organizational abilities compared to healthy controls (HCs). Second, based on the characteristics of OCD, namely, an obsession with detail without seeing the bigger picture, we hypothesized that the range of the eye gaze distribution within the RCFT figure during memorization would reflect the organizational ability of the participants (i.e., a wider range of eye gaze would indicate a better organizational ability than a gaze focusing on only a narrow part of the RCFT figure).

## Results

### Participant characteristics

The demographic and clinical characteristics of the participants are summarized in Table 1. There were more males than females in the OCD group than in the HC group (χ^2^ = 10.806, p = 0.001). The patients with OCD were older (t = 2.629, p = 0.009), less educated (t = − 4.071, p < 0.001), and had a lower intelligence quotient (IQ; t = − 4.713, p < 0.001) than the HC subjects. Handedness and the proportion of participants wearing glasses were not different between the OCD and HC groups.

**Table 1:** Demographic and clinical characteristics of the patients with obsessive–compulsive disorder (OCD) and healthy controls (HCs).

	OCD (N = 104)	HC (N = 114)	Statistical analysis^a^	
			χ^2^ or T	p
Sex (male/female)	73/31	55/59	10.806	0.001**
Handedness (right/left)	97/7	109/5	0.575	0.448
Glasses (yes/no)	51/53	45/69	2.019	0.155
Age (years)	26.2 ± 6.6	24.3 ± 3.5	2.629	0.009*
Education (years)	13.9 ± 2.2	15.0 ± 1.9	−4.071	< 0.001**
IQ	110.9 ± 14.1	119.6 ± 13.2	−4.713	< 0.001**
Duration of illness (years)	8.9 ± 6.2	–	–	–
Age of onset (years)	17.3 ± 6.0	–	–	–
**Y-BOCS scores**				
Total	15.5 ± 6.3	0.6 ± 1.9	24.069	< 0.001**
Obsession	7.9 ± 3.4	0.2 ± 1.0	22.897	< 0.001**
Compulsion	7.6 ± 3.6	0.3 ± 1.1	20.970	< 0.001**
HAM-D score	6.4 ± 3.7	3.6 ± 2.7	6.437	< 0.001**
HAM-A score	5.6 ± 2.9	2.9 ± 2.2	7.721	< 0.001**
**Main symptom category**^**b**^				
Contamination and cleaning	30 (28.8)	–	–	–
Hoarding and collecting	0 (0.0)	–	–	–
Symmetry, ordering, counting and arranging	12 (11.5)	–	–	–
Harm due to injury, violence, aggression	27 (26.0)	–	–	–
Sexual and religious	4 (3.8)	–	–	–
Miscellaneous	31 (29.8)	–	–	–
Comorbidity^c^				
None	55 (52.9)	–	–	–
Depressive disorder	16 (15.4)	–	–	–
Bipolar disorder	15 (14.4)	–	–	–
Anxiety disorder	7 (6.7)	–	–	–
Personality disorder	6 (5.8)	–	–	–
Miscellaneous	13 (12.5)	–	–	–
**Medication**^**d**^				
None	5 (4.8)	–	–	–
Antidepressant	97 (93.3)	–	–	–
Anxiolytics	58 (55.8)	–	–	–
Antipsychotics	55 (52.9)	–	–	–
Mood stabilizers	17 (16.3)	–	–	–

*IQ* intelligence quotient, *Y-BOCS* Yale-Brown Obsessive Compulsive Scale, *HAM-D* Hamilton Rating Scale for Depression, *HAM-A* Hamilton Rating Scale for Anxiety.

*The mean difference is significant at the 0.05 level.

**The mean difference is significant at the 0.005 level.

^a^Independent t test or Welch's t test if the variances were not equal and χ2 analysis or Fisher's exact test for categorical data.

^b^Number (percentage) of patients who showed each main symptom on the dimensional Y-BOCS.

^c^Number (percentage) of patients who were diagnosed with each comorbid psychiatric disorder. For depressive disorder, 16 patients were diagnosed with depressive disorder, not otherwise specified (NOS). For bipolar disorder, 2 patients were diagnosed with bipolar I disorder, 12 with bipolar II disorder, and 1 with bipolar disorder, NOS. For anxiety disorder, 6 patients were diagnosed with panic disorder and 1 with anxiety disorder, NOS. For personality disorder, 2 patients were diagnosed with schizotypal personality disorder, 3 with obsessive–compulsive personality disorder, and 1 with avoidant personality disorder. In the miscellaneous group, 7 patients were diagnosed with trichotillomania, 3 with tic disorder, 2 with Tourette's disorder, and 1 with adjustment disorder.

^d^Number (percentage) of patients who were prescribed each medication.

Data are given as the mean ± standard deviation.

### RCFT and eye tracking results

**Figure 1:**
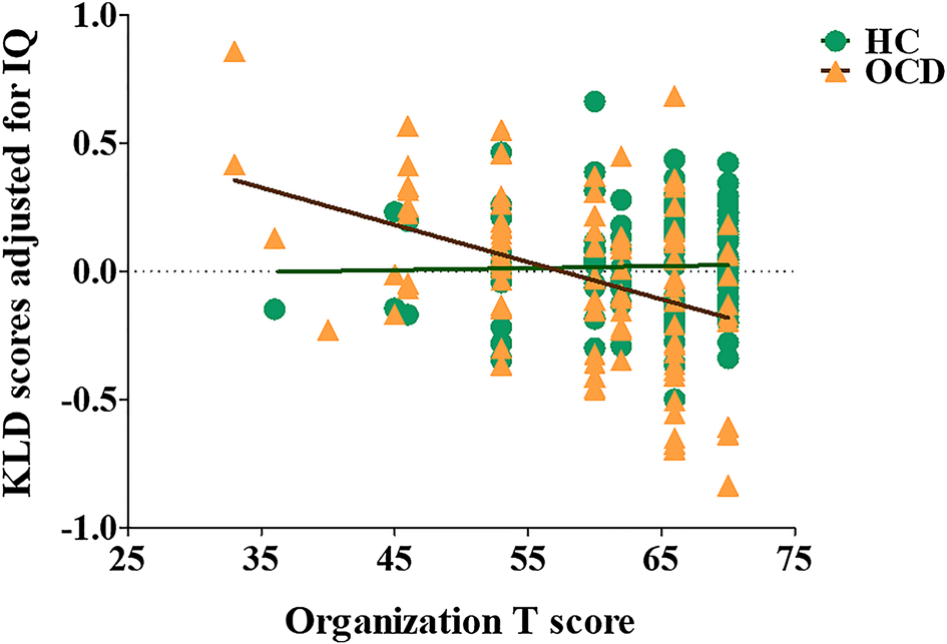
Correlation analysis between the Kullback–Leibler divergence (KLD) scores adjusted for intelligence quotient (IQ) and the organization T scores in obsessive–compulsive disorder (OCD) patients (r = − 0.405, p < 0.001) and healthy control (HC) subjects (r = 0.032, p = 0.739).

Analysis of covariance (ANCOVA) using education years and IQ as covariates revealed that the patients with OCD had lower immediate recall T scores (F = 6.733, p = 0.010) and organization T scores (F = 14.893, p < 0.001) compared to HCs. ANCOVA using age, sex, education years, and IQ as covariates showed that OCD patients had lower organization total scores (F = 7.810, p = 0.006) and planning scores (F = 6.050, p = 0.015) compared to HCs. Other variables regarding RCFT performance did not differ between the OCD and HC groups (Table 2). Kullback–Leibler divergence (KLD) scores were calculated from the eye tracking data to evaluate the distance between a participant’s eye gaze distribution and a hypothetical uniform distribution within the RCFT figure. Narrower gaze distributions within the RCFT figure (i.e., higher KLD scores) indicated that the participant was more obsessed with detail and had less organizational strategy. ANCOVA using age, sex, education years, and IQ as covariates revealed no significant group differences in KLD scores between OCD patients and HCs (F = 3.662, p = 0.057; Table 2). Pearson’s correlation analysis with IQ as a covariate showed a significant negative correlation between the KLD scores and organization T scores in the OCD patients (r = − 0.405, p < 0.001) but not in the HC subjects (r = 0.032, p = 0.739; Fig. 1).

**Table 2:** Comparison of the Rey–Osterrieth complex figure test (RCFT) results across the patients with obsessive–compulsive disorder (OCD) and healthy controls (HCs).

	OCD (N = 104)	HC (N = 114)	Statistical analysis^a^	
			F	p
Immediate recall time (s)	118.5 ± 46.3	117.7 ± 50.2	0.699	0.404
Immediate recall T score	55.7 ± 10.5	61.4 ± 9.4	6.733	0.010*
Immediate recall total score	13.7 ± 3.4	15.3 ± 2.7	0.047	0.828
Configural presence score	4.4 ± 6.9	3.9 ± 0.3	0.983	0.323
Configural accuracy score	2.8 ± 1.0	3.0 ± 0.8	0.000	0.989
Cluster presence score	3.2 ± 0.9	3.5 ± 0.7	0.006	0.941
Cluster accuracy score	1.9 ± 1.0	2.3 ± 1.0	0.544	0.461
Detail presence score	2.2 ± 1.1	2.6 ± 1.1	0.119	0.730
Organization T score	59.0 ± 8.7	63.4 ± 7.3	14.893	< 0.001**
Organization total score	6.8 ± 1.2	7.3 ± 0.9	7.810	0.006*
Fragmentation score	3.5 ± 0.6	3.7 ± 0.5	3.788	0.053
Planning score	3.3 ± 1.0	3.6 ± 0.7	6.050	0.015*
Kullback–Leibler divergence score	2.5 ± 0.3	2.6 ± 0.2	3.662	0.057

Data are given as the mean ± standard deviation.

*The mean difference is significant at the 0.05 level.

**The mean difference is significant at the 0.005 level.

^a^Analysis of covariance (ANCOVA) using education years and intelligence quotient (IQ) as covariates for group comparisons of T scores. ANCOVA using age, sex, education years, and IQ as covariates for group comparisons of other variables.

## Discussion

In the current study, we aimed to develop direct, simple, and rapid measurements of organizational abilities during visual encoding processes in patients with OCD using a newly developed eye tracking-based RCFT. Behavioral performances on the RCFT showed that the patients with OCD had significantly poorer scores on immediate recall and organizational abilities than HCs. In addition, KLD scores, which reflect the distribution of gaze during memorization of the RCFT figure, were associated with organizational abilities in the OCD group but not in the HC group. The current study findings suggest that eye tracking biomarkers of visual memory encoding provide rapidly determined indexes of executive functioning in patients with OCD.

In line with previous studies, we found that patients with OCD showed poorer performance in the RCFT immediate recall condition than HCs^18–21^. In addition, impaired organizational abilities found in OCD patients in the current study support previous study results that organization dysfunction during memorization of the RCFT figure mediated impaired immediate recall performance in patients with OCD^11–13,22^. Because IQ was controlled in all statistical analyses in this study, impaired organizational abilities of OCD patients were not the consequences of general cognitive impairments, but were more closely related to dysfunctions in executive or strategic aspects of visual memory encoding. Therefore, the current study population is representative of individuals with impaired executive or strategic aspects of memory (i.e., planning and organizational process), which have been related to frontostriatal system dysfunction and a neurocognitive characteristic of OCD^23^.

In addition, we found that higher KLD scores (i.e., narrower gaze distribution within the RCFT figure) were associated with poorer organizational T scores measured at the behavioral level in the OCD patients. This result indicated that OCD patients with poor executive functioning show abnormal eye movement characteristics, such as an obsession with detail without seeing the entire picture while memorizing the RCFT figure. On the other hand, eye movement patterns were not correlated with executive functioning in the HCs, suggesting that this association may be evident only within patient groups with neurobiological abnormalities. These findings support frontostriatal system dysfunction as a neurobiological underpinning of OCD related to impaired executive functioning^11,14^. In addition, KLD scores reflecting eye gaze characteristics during visual encoding processes could be used as a simple and rapidly determinable biomarker of frontostriatal dysfunction of OCD patients that has advantages in clinical utility.

This study has several limitations. First, almost every patient with OCD was being treated with a selective serotonin reuptake inhibitor (SSRI) or cognitive behavioral therapy (CBT) before participating in the eye tracking-based RCFT. Although it has been shown that these types of treatment do not improve RCFT performance^18,20,21^, the effects of SSRIs or CBT on eye movements during visuospatial memory encoding have not yet been sufficiently studied. Second, demographic factors, such as age, sex, education years, and IQ, were not matched between the OCD and HC groups. Thus, the potential confounding effect of these demographic factors should be considered in interpreting the current study results. However, these factors were controlled as covariates, and T scores were used in group comparisons of behavioral performance on the RCFT. Third, because KLD scores calculated from the eye tracking data represent a novel biomarker used for the first time in the current study, the scarce number of related studies limits the interpretation of the group comparison results of KLD scores.

Although the RCFT is a well-validated tool for evaluating executive function (i.e., planning and organizational abilities) during visuospatial memory encoding, time-consuming and complex processes make it difficult to widely use it in actual clinical practice. Moreover, because organizational abilities in the copy condition are evaluated by immediate retrieval after encoding, they are an indirect measure of visual memory encoding and affected by visuomotor function and thus can only provide behavioral level information. In response to the need to develop biomarkers reflecting frontostriatal function that can be measured easily and in a short time, we developed the eye tracking-based RCFT and found eye movement characteristics (i.e., KLD scores) that reflected the organizational abilities of OCD patients during visuospatial memory encoding. This study is the first to provide a potential biological method that could evaluate executive function in OCD patients with only 3 min of eye tracking. Challenges remain in translating the findings of the current study into a clinically feasible frontostriatal function test.

## Methods

### Participants

A total of 146 OCD patients and 150 HC subjects participated in this study. After quality control processing of the eye tracking data, data of 104 OCD patients and 114 HCs were used for the analyses. Patients with OCD were recruited from the OCD clinic at Seoul National University Hospital (SNUH) and fulfilled the Diagnostic and Statistical Manual of Mental Disorders-IV (DSM-IV) criteria for OCD. HC subjects were recruited via an online advertisement. The Yale-Brown Obsessive Compulsive Scale (Y-BOCS)^24^, Hamilton Rating Scale for Depression (HAM-D)^25^, and Hamilton Rating Scale for Anxiety (HAM-A)^26^ were used to evaluate the severity of OCD, depressive and anxious symptoms, respectively. The duration of illness, symptom ratings, main symptom category, comorbid psychiatric disorders, and prescribed medications were evaluated by a certified psychiatrist during a clinical interview. IQs were assessed using the abbreviated version of the Korean-Wechsler Adult Intelligence Scale, which includes four subsets^27^.

Common exclusion criteria for the two groups included a lifetime diagnosis of psychotic disorders, substance abuse or dependence (except nicotine), neurological disease or significant head trauma, medical illness that could be accompanied by psychiatric symptoms, visual impairments, wearing contact lenses, and intellectual disability (IQ < 70).

Written informed consent was received from all participants after providing a thorough explanation of the study procedure. For the minors who participated in this study, informed consent was obtained from both the participants themselves and their caretakers. This study was conducted in accordance with the Declaration of Helsinki and was approved by the institutional review board of SNUH (IRB no. H-1611-112-810).

### RCFT and eye tracking

The RCFT figure was displayed on a 19-inch monitor with dimensions of 1280 × 1024 pixels using Experiment Builder v.2.1.45 software (SR research, Ottawa, Ontario, Canada). The participant’s head rested on a chin rest in a dimly lit room. The distance between the chin rest and the monitor was 70 cm. The horizontal viewing angle was 22°, and the vertical viewing angle was 17°. The participants were instructed to memorize the RCFT figure for 3 min, while their eye movements were recorded using EyeLink 1000 (SR research) at a 1000-Hz sampling rate. The encoding time was decided based on a previous study reporting that the mean response time of the copy condition during the original RCFT was approximately 3 min^18^. Nine-point calibration and verification were performed before measuring eye movements. Immediately after completing the eye tracking, the participants were asked to draw the figure from memory, as in the immediate recall condition of RCFT, and response times were recorded. While the participant drew the RCFT figure from memory, the order in which the participant drew the parts of the RCFT figure was numbered as a flowchart to track the participant’s rendition of the figure to evaluate organizational strategies as a replacement for the RCFT copy condition. RCFT immediate recall performance and organizational ability were scored using the Boston Qualitative Scoring System (BQSS)^28^. Three researchers contributed to eye tracking and RCFT data collection.

### Eye-tracking data analysis

**Figure 2:**
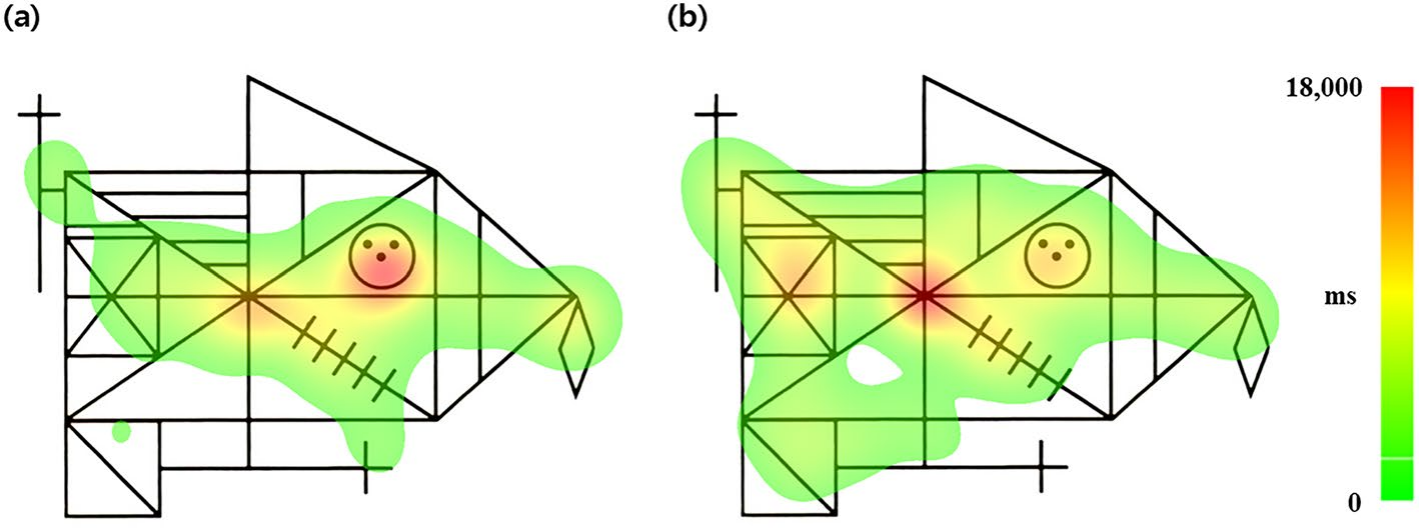
Hit maps showing cumulative eye gaze during the 3 min of memorizing the Rey-Osterrieth Complex Figure Test (RCFT) figure. (**a**) The distribution of eye gaze within the RCFT figure was narrow in a patient with the lowest organization T score (i.e., 33). (**b**) The distribution of eye gaze within the RCFT figure was wide in a patient with the highest organization T score (i.e., 70). The colored bar with numbers indicates the dwelling time of eye gaze (ms).

Eye movement data were analyzed using EyeLink Data Viewer v.2.6.1 (SR research) and customized Python scripts. Two independent researchers (M. Kim and T.H. Lee) who were blinded to the groups performed quality control of the eye tracking data by careful visual inspection. Data of participants whose eye gaze was largely shifted from the RCFT figure or dwelling time outside the RCFT figure was greater than 100 s were excluded from the final analyses. After quality control processing, data of 104 OCD patients and 114 HCs were used for the analyses. Based on the finding that the eye gaze distribution within the RCFT figure was wider in the patient with the highest organization T score (i.e., 70; Fig. 2b) than in the patient with the lowest organization T score (i.e., 33; Fig. 2a), we calculated the KLD score to evaluate the distance between a participant’s eye gaze distribution and a hypothetical uniform distribution within the RCFT figure^29^. For discrete probability distributions P and Q, KLD is calculated as follows:

$${D}_{KL}\left(\mathrm{P}\parallel {\mathrm{Q}}\right)= \sum_{x\in \chi }P\left(x\right){\mathrm{log}}\left(\frac{P\left(x\right)}{Q\left(x\right)}\right)$$

where P represents the true distribution (i.e., uniform distribution in our study) and Q represents one distribution (i.e., participant’s eye gaze distribution). The KLD is a nonnegative measure, and ${D}_{KL}\left(\mathrm{P}\parallel \mathrm{Q}\right)=0$ if and only if $P=Q$. Computing the KLD has some difficulty when $Q\left(i\right)=0 \mathrm{and} P(i)\ne 0$. According to the above definition, the KLD goes to ∞ when $Q\left(i\right)=0 \mathrm{and} P(i)\ne 0$. Therefore, we used the “reverse” KLD, which is calculated as follows:

$${D}_{KL}\left({\mathrm{Q}}\parallel \mathrm{P}\right)= \sum_{x\in \chi }Q\left(x\right){\mathrm{log}}\left(\frac{Q\left(x\right)}{P\left(x\right)}\right)$$

If the participant looked at the RCFT figure evenly, the KLD score would approach 0. Narrower gaze distributions within the RCFT figure (i.e., the higher KLD score) indicate that the participant was more obsessed with detail without seeing the bigger picture.

### Statistical analysis

Statistical analyses were performed using SPSS v.25.0 (IBM, Armonk, NY, USA), and statistical significance was set at p < 0.05. The demographic and clinical characteristics were compared using independent t tests across the groups for the continuous variables. Chi-square tests were used for the categorical variables. ANCOVA using education years and IQ as covariates was performed for the group comparison of immediate recall T scores and organization T scores. For the group comparison of other RCFT scores and KLD scores, ANCOVA using age, sex, education years, and IQ as covariates was performed. Partial correlation analysis with IQ as a covariate was used to assess the relationship between KLD scores and RCFT organization T scores.

## Data Availability

The data that support the findings of this study are available from the corresponding author upon reasonable request.
